# Understanding Gangrene in the Context of Peripheral Vascular Disease: Prevalence, Etiology, and Considerations for Amputation-Level Determination

**DOI:** 10.7759/cureus.49026

**Published:** 2023-11-18

**Authors:** Abhilasha Bhargava, Chandrashekhar Mahakalkar, Shivani Kshirsagar

**Affiliations:** 1 General Surgery, Jawaharlal Nehru Medical College, Datta Meghe Institute of Higher Education and Research, Wardha, IND

**Keywords:** early intervention, multidisciplinary care, limb salvage, ischemia, peripheral vascular disease, gangrene

## Abstract

Gangrene is a grave complication of peripheral vascular disease (PVD), characterised by tissue necrosis due to inadequate blood supply. This review article comprehensively explores gangrene in PVD, encompassing its prevalence, aetiology, clinical presentation, diagnostic modalities, management strategies, prognosis, and future directions. Key factors influencing outcomes, including the timeliness of intervention and the choice between limb salvage and amputation, are identified. Moreover, this review underscores the importance of early detection and multidisciplinary care, emphasising the significance of patient-centred approaches. It also calls for increased awareness, continued research, and innovative solutions to improve the lives of individuals grappling with gangrene in the context of PVD.

## Introduction and background

Peripheral vascular disease (PVD), often referred to as PVD, is a prevalent condition that affects blood vessels outside the heart and brain, primarily involving the arteries and veins in the extremities. It significantly hampers the vascular system's capacity to supply oxygen and essential nutrients to tissues, particularly in the limbs. Atherosclerosis stands as the most common underlying cause of PVD [1]. This condition manifests in a wide array of clinical forms, ranging from intermittent claudication, which induces pain during physical activities, to critical limb ischemia, a severe state that may culminate in limb loss [2].

Gangrene, on the other hand, is a pathological state characterized by tissue death and decomposition due to a lack of blood supply. It serves as a severe and potentially life-threatening manifestation of various underlying medical conditions, primarily linked to vascular insufficiency. The term "gangrene" encompasses different forms of tissue necrosis, including dry, wet, and gas gangrene, each presenting distinct clinical features and posing unique management challenges [1].

The principal objective of this review article is to offer an all-encompassing perspective on the intricate relationship between gangrene and peripheral vascular disease. We intend to explore the prevalence, causative factors, clinical manifestations, management approaches, prognosis, and potential future directions concerning gangrene within the context of PVD. By amalgamating existing research and the latest developments in the field, this article endeavors to serve as an invaluable resource for healthcare professionals, researchers, and anyone keen on comprehending the complexities of gangrene in vascular pathology. We aspire to illuminate the critical considerations for healthcare providers and underscore the significance of early diagnosis and timely intervention in the treatment of gangrene in PVD patients. Through this comprehensive review, we aim to contribute to enhanced patient outcomes and the advancement of innovative approaches in the realm of vascular medicine.

## Review

Prevalence of gangrene in PVD

Epidemiological Data

Epidemiological data are essential to understand the scope and impact of gangrene in PVD. The one-year cumulative incidence of gangrene in peripheral artery disease (PAD) is 16.7%. PAD is one of the top risk factors for gangrene [3]. Studies and statistics related to the prevalence of gangrene within the PVD population provide valuable insights into the burden of this condition. Such data not only assist in assessing the scale of the problem but also aid in resource allocation, healthcare planning, and preventive measures [3,4].

Epidemiological research has revealed that gangrene is a significant clinical complication of PVD, with varying prevalence rates depending on geographic location, population demographics, and risk factors. The incidence of gangrene increases with age, and individuals with comorbidities such as diabetes, hypertension, and smoking history are at higher risk [3].

Risk Factors

Identifying and understanding the risk factors associated with gangrene in PVD is crucial for clinical management and prevention efforts. Several factors contribute to an individual's susceptibility to developing gangrene in the context of PVD. These risk factors encompass both modifiable and non-modifiable elements, including the following.

Atherosclerosis: Atherosclerosis is a primary and pervasive underlying cause of PVD. This pathological process involves the gradual buildup of atherosclerotic plaques within arterial walls. These plaques narrow and harden the arteries, leading to diminished blood flow to the extremities. As a result, tissues become vulnerable to ischemia, setting the stage for the development of gangrene [5].

Diabetes mellitus: Diabetic vasculopathy is a well-established risk factor for gangrene in individuals with PVD. Chronic hyperglycemia, a hallmark of diabetes, takes a toll on blood vessels, leading to endothelial dysfunction and accelerated atherosclerosis. Additionally, diabetes can impair wound healing and reduce the body's ability to fight infections, thus increasing the likelihood of gangrene development [6].

Smoking: Smoking remains a significant contributor to PVD and is strongly associated with an increased risk of gangrene. The harmful chemicals in tobacco smoke, such as nicotine, carbon monoxide, and tar, harm blood vessels. Smoking promotes inflammation, constricts blood vessels, and encourages the formation of atherosclerotic plaques, making smokers significantly more vulnerable to gangrene [7].

Hypertension: High blood pressure is another modifiable risk factor that accelerates the progression of PVD and raises the risk of gangrene. Elevated blood pressure can further damage already compromised blood vessels, exacerbating atherosclerosis and increasing the likelihood of thromboembolic events, which can precipitate ischemia [8].

Hyperlipidemia: Elevated cholesterol levels in the bloodstream contribute to the development and progression of atherosclerosis. As cholesterol accumulates within arterial walls, it fosters plaque formation and narrowing of blood vessels, perpetuating the cycle of reduced blood flow, tissue ischemia, and susceptibility to gangrene [9].

Obesity: Excess body weight and obesity significantly strain the circulatory system. The additional adipose tissue requires a more excellent blood supply, which can exacerbate the effects of PVD. Obesity amplifies the risk of developing gangrene by intensifying the burden on already compromised blood vessels [10].

Impact on the Healthcare System

Prolonged hospital stays: Patients with gangrene often require extended hospitalisation due to the complexity of their condition. The management of gangrene necessitates close monitoring, specialised wound care, and often multiple interventions, all of which contribute to prolonged hospital stays. These extended stays strain hospital resources, including bed availability, staffing, and healthcare costs [11].

Surgical interventions: In many cases, surgical interventions are required to address gangrene effectively. These procedures may include debridement to remove necrotic tissue, revascularisation techniques to restore blood flow, or, in severe cases, amputation. Surgical interventions involve operating room time, surgical teams, and post-operative care, adding to the healthcare system's workload and expenses [12].

Specialized wound care: Gangrene wounds demand specialised and often long-term care. This care includes using advanced wound dressings, frequent dressing changes, and close monitoring for signs of infection or deterioration. Skilled healthcare providers, such as wound care specialists and nurses, are required to provide this level of care, increasing the healthcare system's workload [13].

Amputation: In cases where gangrene is extensive or uncontrolled, amputation may be necessary. Amputation procedures are complex, requiring surgical expertise and post-operative rehabilitation. The long-term care of amputees also involves prosthetic fitting and rehabilitation services, adding to the healthcare system's responsibilities [14].

Economic costs: The economic costs of managing gangrene are substantial. These costs encompass direct medical expenses, such as hospitalisation, surgeries, medications, and wound care supplies, and indirect costs related to the loss of productivity. Patients with gangrene may face extended periods of disability, making them unable to work or contribute to their households. This loss of productivity has economic repercussions on both the individual and society [15].

Resource allocation: The management of gangrene necessitates allocating healthcare resources, including hospital beds, surgical facilities, and specialised medical personnel. The demand for these resources can strain the capacity of healthcare systems, potentially affecting the care provided to other patients [16].

Etiology of gangrene in PVD

Ischemia as a Primary Cause

Atherosclerosis: Atherosclerosis contributes to ischemic gangrene in PVD. This progressive disease leads to the formation of atherosclerotic plaques within arterial walls, causing narrowing and obstruction of blood vessels. In advanced stages, these plaques can rupture, leading to thrombosis (blood clot formation) and acute occlusion of arteries. The resultant lack of blood flow deprives tissues of oxygen and nutrients, predisposing them to ischemic injury and gangrene [17].

Thromboembolic events: Thromboembolic events, such as embolism or thrombosis, can suddenly occlude arteries downstream, causing acute ischemia and, potentially, gangrene. Emboli can originate from various sources, including atherosclerotic plaques or cardiac sources such as atrial fibrillation, while thrombosis may develop within diseased arteries [18].

Diabetic vasculopathy: Diabetic vasculopathy, a complication of diabetes mellitus, significantly contributes to ischemic gangrene. Chronic hyperglycemia damages blood vessels, promoting atherosclerosis and microvascular abnormalities. Diabetic neuropathy can also reduce pain perception, delaying the recognition of ischemia and worsening tissue damage [19].

Infection as a Contributing Factor

Bacterial infection: Bacterial infections are frequently encountered in gangrenous tissue. The devitalised and necrotic tissue from compromised blood supply provides an ideal breeding ground for bacteria. The absence of adequate blood flow limits the body's ability to mount an effective immune response, allowing bacteria to proliferate. This unchecked bacterial growth can lead to a spectrum of complications, including cellulitis, the formation of abscesses, and the potential for systemic sepsis if left untreated. Cellulitis is a spreading skin infection characterised by redness, warmth, pain, and swelling, often surrounding the gangrenous site. Abscess formation represents localised pockets of pus within the affected tissue, signifying a more severe infection. If bacteria from gangrenous tissue enter the bloodstream, it can result in systemic sepsis, a life-threatening condition requiring immediate medical intervention [1].

Fungal infection: In specific instances, notably with wet gangrene or gangrene involving genital areas, fungal infections can take hold. Fungi thrive in moist and warm environments, making gangrenous tissue, often necrotic and damp, an ideal habitat for fungal growth. These fungal infections can further exacerbate tissue necrosis and complicate the clinical picture. Managing fungal gangrene requires specialised antifungal treatments and meticulous wound care [20].

Role of Trauma and Complications

Trauma: Traumatic injuries, whether they result from cuts, burns, or pressure ulcers, can significantly exacerbate the progression of gangrene. These injuries often occur in areas already compromised due to reduced blood flow. When trauma disrupts the fragile balance of oxygen and nutrient supply to tissues already struggling with ischemia, it can accelerate tissue necrosis. For instance, a minor cut or abrasion in a PVD-affected limb can quickly escalate into a non-healing ulcer, providing a gateway for infection to set in. Pressure ulcers, common in individuals with limited mobility, create localised areas of tissue damage due to sustained pressure, particularly over bony prominences. These ulcers, if left unattended, can become gangrenous, adding to the complexity of the clinical picture [21].

Complications: Surgical procedures aimed at addressing PVD, such as bypass surgery or the placement of stents, can also introduce complications that contribute to the development or exacerbation of gangrene. For instance, graft failure in bypass surgery can lead to the restoration of blood flow not being sustained, resulting in persistent ischemia. Similarly, thrombosis or restenosis (re-narrowing of the artery) in stents can impede blood flow, undermining the intended therapeutic effect. These complications, if not promptly identified and managed, can culminate in the progression of gangrene [22].

Clinical presentation and diagnosis of gangrene in PVD

Signs and Symptoms

In the PVD context, gangrene presents diverse signs and symptoms, each offering valuable clinical cues that are pivotal for timely diagnosis and intervention. Recognising these indicators is paramount, as they are early warning signs, facilitating prompt medical attention. Common signs and symptoms associated with gangrene in PVD encompass the following:

Pain: One of the hallmark symptoms of gangrene is pain, often severe, throbbing, or burning. This pain typically emanates from the affected extremity and can be debilitating. Significantly, the pain may worsen with physical activity or movement, known as intermittent claudication. Patients often describe this as discomfort or cramping in the leg muscles during exertion [23].

Pallor: Reduced blood flow to the affected limb leads to a noticeable pallor or paleness in the skin. This pallor is especially apparent when the limb is elevated. In some cases, the difference in colour between the affected and unaffected extremities can be striking as a visual indicator of compromised blood circulation [24].

Coolness: Gangrenous tissue often exhibits a distinct coolness compared to the surrounding healthy tissue - the diminished blood flow results in a lower temperature within the affected area. Patients and healthcare providers can detect this temperature difference by touch [25].

Loss of sensation: Peripheral neuropathy is a common occurrence in PVD patients, and it frequently leads to a reduction or complete loss of sensation in the affected limb. This sensory impairment can mask pain and make it challenging for patients to recognise early signs of gangrene. Diminished sensation underscores the importance of routine foot inspections and vigilance in detecting abnormalities [26].

Ulceration: Gangrene frequently progresses to the point of skin breakdown, forming open sores or ulcers. These ulcers often develop in areas subjected to pressure, particularly over bony prominences. These wounds are often slow to heal and are vulnerable to infection, further complicating the clinical picture [27].

Gangrenous odor: In advanced cases of gangrene, a characteristic foul odour may emanate from the affected area. This odour is a result of the presence of necrotic tissue and associated bacterial or fungal infection. It can be distressing for patients and is a sign of advanced tissue decay [28].

Diagnostic Modalities

Angiography: Angiography involves injecting a contrast dye into the blood vessels and taking X-ray images. It helps visualise the extent of arterial blockages, narrowing, or occlusions. This diagnostic technique is invaluable in identifying areas of reduced blood flow that may contribute to gangrene [29].

Doppler ultrasound: Doppler ultrasound is a non-invasive imaging technique that uses sound waves to evaluate blood flow. It can detect abnormalities in blood vessel structure and flow patterns. Doppler ultrasound is beneficial for assessing peripheral arterial disease (PAD) and identifying areas of reduced blood flow [30].

Laboratory Tests

Inflammatory markers: Blood tests, such as C-reactive protein (CRP) and erythrocyte sedimentation rate (ESR), can be elevated in the presence of infection or inflammation associated with gangrene. Elevated levels of these markers may indicate the need for further investigation and intervention [31].

Cultures: If infection is suspected, tissue cultures (e.g., wound cultures) may be obtained to identify the causative microorganisms and guide antibiotic therapy. Culture results help tailor treatment to the specific pathogens involved [32].

Staging and Grading Systems

Rutherford classification: The Rutherford classification system is a widely used tool for categorising PAD, a common component of PVD, into distinct stages based on clinical presentation and severity. This system acknowledges a range of symptoms and complications, including intermittent claudication (pain during physical activity), rest pain (pain at rest), ulceration, and gangrene. The Rutherford classification comprises stages 0 to 6, each reflecting increasing severity and clinical complexity. Stage 6 corresponds to the most advanced cases, where significant tissue loss or gangrene necessitates urgent intervention [33].

Wagner classification: The Wagner classification system assesses diabetic foot ulcers, which frequently precede or accompany gangrene in individuals with diabetic PVD. This system categorises diabetic foot ulcers into grades 0-5, each corresponding to a different severity level and tissue involvement. Grade 0 signifies no ulceration, while grade 5 indicates extensive gangrene affecting the entire foot. The Wagner classification aids healthcare providers in determining appropriate interventions and evaluating the progression of diabetic foot complications [34].

Fontaine classification: The Fontaine classification system is another valuable tool for categorising PAD into stages, ranging from 1 to 4. This classification is based on the evaluation of symptoms and functional impairment. Stage 1 represents early disease with minimal symptoms, while stage 4 signifies advanced disease, including gangrene. This system helps healthcare professionals gauge the severity of PVD, determine treatment strategies, and assess the impact on the patient's overall functional status [35].

Management of gangrene in PVD

Conservative Treatment Options

Wound care and dressings: Wound care represents a cornerstone of gangrene management in PVD patients. It involves meticulous attention to the affected area, primarily focusing on cleaning and debridement of necrotic tissue. Debridement is crucial as it removes non-viable tissue, creating a healthier wound bed conducive to healing. Dressings, such as hydrogels or alginate dressings, are frequently employed. These dressings maintain a moist wound environment, which removes dead tissue and promotes tissue regeneration. Appropriate dressings facilitate healing, help prevent infection, and minimise pain associated with exposed nerve endings [11].

Antibiotics: Antibiotic therapy is critical when infection is present or suspected within gangrenous tissue. Infection can significantly complicate gangrene, necessitating prompt intervention. Empirical antibiotics are often initiated while awaiting culture results to cover a broad spectrum of potential pathogens. Once culture results become available, antibiotic therapy can be adjusted to target specific microorganisms responsible for the infection. The selection of antibiotics should be guided by the susceptibility profile of the identified pathogens, ensuring the most effective treatment regimen. The administration of antibiotics aims not only to control infection but also to prevent its systemic spread, reducing the risk of sepsis [36].

Pain management: Managing pain is an integral aspect of caring for individuals with gangrene, as they often experience severe discomfort due to tissue ischemia, inflammation, and nerve involvement. Effective pain relief is essential to improve a patient's quality of life and well-being. Pain management strategies may encompass various analgesics, including opioids for severe pain, non-steroidal anti-inflammatory drugs (NSAIDs) for inflammation control, and neuropathic pain medications for pain of neuropathic origin. Multimodal approaches that combine medications with non-pharmacological interventions, such as physical therapy and psychological support, can effectively address the complex pain associated with gangrene [37].

Revascularization Techniques

Revascularisation techniques are a critical component of the therapeutic arsenal in managing gangrene within PVD. These interventions aim to restore or improve blood flow to the affected limb, addressing the underlying ischemia and promoting tissue viability. Two primary revascularisation techniques employed in the management of gangrene in PVD patients are angioplasty with stenting and bypass surgery:

Angioplasty and stenting: Angioplasty is a minimally invasive procedure to alleviate arterial stenosis or blockage. During this procedure, a balloon-tipped catheter is advanced into the narrowed or obstructed artery, which inflates the balloon. The inflation exerts pressure against the arterial plaque, widening the vessel lumen and restoring blood flow. This technique is highly effective in improving circulation to the affected limb. In some cases, stents, which are metallic mesh tubes, may be deployed within the treated segment to provide structural support and maintain vessel patency. Drug-eluting stents, coated with medications that inhibit restenosis (re-narrowing of the artery), are also utilised to enhance long-term results. Angioplasty and stenting are minimally invasive, requiring smaller incisions and shorter recovery times than traditional surgery [38].

Bypass surgery: Bypass surgery, also known as arterial revascularisation, is a surgical procedure that involves creating a detour or bypass around a blocked or severely narrowed segment of an artery. This is achieved using a graft, typically harvested from another part of the patient's body (autologous graft) or constructed from synthetic material (synthetic graft). The graft is surgically attached to the artery above and below the blocked area, creating a new pathway for blood to flow. Bypass surgery aims to restore adequate blood flow to the affected limb, bypassing the obstructed segment. It is often considered when angioplasty is not feasible due to the location or extent of arterial disease or when previous angioplasty has been ineffective. Bypass surgery is particularly beneficial in extensive arterial blockages or multi-level occlusions [39].

Amputation Considerations

Amputation is a profound and often last-resort intervention in managing gangrene within the context of PVD. When conservative treatments and revascularisation techniques prove inadequate to salvage the affected limb or when severe infection or gangrene threatens the patient's life, amputation becomes necessary.

Indications for amputation: The decision to proceed with amputation hinges on carefully evaluating clinical indications. It is typically reserved for situations where conservative therapies have failed, or limb viability is severely compromised. Common indications include uncontrolled sepsis, characterised by gangrene-associated infections resistant to antibiotic therapy and leading to systemic sepsis. Additionally, extensive tissue necrosis may necessitate amputation, rendering a significant portion of the limb non-viable. In cases where revascularisation is impossible or offers a dismal prognosis for functional recovery, amputation may be the only viable option. Finally, amputation is considered when the patient experiences intractable pain that severely impairs their quality of life, with no relief achievable through alternative interventions [40].

Levels of amputation: The choice of the level of amputation is a crucial decision influenced by several factors. These factors include the extent and location of gangrene, the potential for functional recovery, and the overall health and preferences of the patient. Depending on these considerations, various levels of amputation may be chosen. Toe amputation, for instance, involves the removal of affected toes while preserving the rest of the foot. Below-knee amputation (BKA) is an option when gangrene primarily affects the lower leg, preserving the knee joint for potential prosthetic fitting. Above-knee amputation (AKA) may be necessary when gangrene extends higher on the limb. Hip disarticulation might be considered in more extensive cases involving removal at the hip joint [41].

Surgical Techniques

The choice of surgical technique for amputation in managing gangrene within PVD is a critical decision influenced by multiple factors, including the level of the amputation and the individual patient's unique circumstances. These surgical techniques are tailored to achieve the best possible functional outcomes while preserving the patient's overall quality of life:

Guillotine amputation: An initial amputation with delayed wound closure is an initial amputation. This technique is typically employed when there is a high degree of infection or uncertainty about the extent of tissue viability. During the initial procedure, the surgeon removes the affected limb up to the designated level of amputation, but the wound is not closed. Instead, it is left open to further assessment, debridement, and infection management. Once the surgeon determines that the infection is under control and the wound is suitable for closure, a definitive surgical closure is performed. Guillotine amputation provides the advantage of immediate infection control and enables a more precise determination of the level of tissue viability before final closure, reducing the risk of complications [42].

Primary closure: Primary closure is preferred in cases with favourable clinical conditions. This technique involves the immediate closure of the surgical wound post-amputation. Primary closure is considered when the surgical site is free from infection, the tissue is healthy, and there are no concerns about compromised wound healing. The primary closure technique offers the benefit of a more rapid healing process and minimises the risk of secondary infection. It is commonly employed in BKAs and toe amputations when the tissue is relatively healthy [43].

Complex surgical reconstructions: In select situations, particularly when preserving limb length and optimising prosthetic fitting are essential, more intricate surgical procedures known as myoplastic or osteoplastic techniques may be considered. These complex reconstructions involve reshaping and repositioning muscles and bones to create a functional residual limb. Myoplastic techniques often entail transferring a muscle flap to improve soft tissue coverage and enhance the prosthetic interface. Osteoplastic procedures may involve bone shortening or reshaping to optimise prosthetic alignment and weight-bearing capabilities. These advanced techniques require specialised expertise and are typically reserved for cases where the preservation of limb length and function is paramount [44].

Multidisciplinary Approach

The management of gangrene in individuals with PVD necessitates a comprehensive and multifaceted approach that extends beyond the expertise of a single healthcare provider. A multidisciplinary approach is essential to address this condition's diverse and complex aspects, preserve the affected limb, achieve functional recovery, and enhance the patient's overall quality of life.

Collaborative team: A multidisciplinary team typically comprises various healthcare professionals with specialised expertise in gangrene management. This team may include vascular surgeons, wound care specialists, infectious disease experts, pain management teams, physical and occupational therapists, and prosthetists. Each team member plays a crucial role in evaluating, treating, and rehabilitating individuals affected by gangrene in the context of PVD [45].

Comprehensive evaluation: The collaborative nature of a multidisciplinary team allows for a comprehensive evaluation of the patient's condition. Vascular surgeons assess the extent and severity of vascular disease, determining the feasibility of revascularisation procedures. Wound care specialists focus on wound assessment, debridement, and selecting appropriate dressings to promote healing. Infectious disease experts guide the management of infections, ensuring the effective use of antibiotics and addressing systemic concerns. Pain management teams work to alleviate the often severe pain associated with gangrene through medications and non-pharmacological approaches. Physical and occupational therapists are involved in rehabilitation, helping patients regain mobility and independence. Prosthetists contribute to prosthetic fitting and alignment for individuals who undergo amputation [46].

Tailored treatment and rehabilitation: Collaboration among these healthcare professionals ensures that treatment plans are tailored to each patient's needs and goals. For some patients, limb salvage through revascularisation techniques may be the primary objective, with vascular surgeons leading the way. Others may require wound care and antibiotic therapy to control infections and promote tissue healing. Physical and occupational therapists work on improving strength, mobility, and functionality, while prosthetists play a critical role in optimising prosthetic fit and function for those who undergo amputation [47].

Optimising outcomes: The multidisciplinary approach optimises outcomes at various levels. It enhances the chances of successful limb salvage by addressing the multiple factors contributing to gangrene. It facilitates functional recovery by providing specialised rehabilitation and prosthetic services. Significantly, it improves the patient's overall quality of life by holistically addressing pain, infection, and mobility issues [48].

Prognosis and outcomes

Factors Influencing Outcomes

Prognosis in cases of gangrene within the context of PVD is influenced by a constellation of factors that collectively shape the trajectory of the disease and the patient's ultimate outcomes. Understanding these determinants is essential for both healthcare providers and patients alike:

Timeliness of intervention: Perhaps the most critical factor influencing outcomes is the timeliness of intervention. Early diagnosis and swift initiation of treatment substantially enhance the chances of successful outcomes. On the other hand, delayed intervention can lead to irreversible tissue damage, increased morbidity, and a decreased likelihood of limb salvage. Timely detection and intervention are essential for preventing the progression of gangrene to advanced stages [49].

Tissue necrosis: The extent of tissue necrosis and the area affected by gangrene is pivotal in determining outcomes. Smaller, well-defined areas of gangrene may be more amenable to conservative treatments, such as wound care and antibiotics or limited amputations. Conversely, extensive tissue involvement may necessitate more radical interventions, such as AKAs, and can significantly impact the patient's functional status post-treatment [42].

Underlying health conditions: Coexisting medical conditions, such as diabetes, cardiovascular disease, renal insufficiency, and immunosuppression, can profoundly complicate the management of gangrene and influence overall prognosis. Patients with these comorbidities often require more comprehensive care and meticulous attention to their health needs to optimise outcomes. Diabetes is a significant risk factor for gangrene due to its detrimental effects on blood vessels and wound healing [50].

Infection control: Successful management of infections associated with gangrene is a cornerstone of improving outcomes. Inadequately controlled infections can lead to systemic spread, sepsis, and a significantly higher mortality risk. Effective antibiotic therapy targeted at the specific pathogens involved, coupled with meticulous wound care and debridement, is essential to prevent the propagation of infection and to facilitate tissue healing [51].

Revascularization success: For cases where revascularisation procedures, such as angioplasty, stenting, or bypass surgery, are viable options, the success of these interventions is a pivotal determinant of outcomes. Adequate restoration of blood flow to the affected limb is crucial for limb salvage and functional recovery. The effectiveness of revascularisation procedures hinges on factors such as the location and severity of arterial blockages and the patient's overall vascular health [52].

Limb Salvage vs. Amputation

The decision between limb salvage and amputation is a pivotal and intricate aspect of managing gangrene within the context of PVD. This decision is not one-size-fits-all but depends on various factors, including the extent of gangrene, infection, the patient's overall health, and functional goals. It is a decision that profoundly impacts a patient's mobility, independence, and quality of life [53].

Limb Salvage

Limb salvage is the preferred outcome whenever it is medically feasible and aligns with the patient's goals. This approach aims to preserve the patient's natural limb, allowing them to maintain mobility and quality of life. Successful limb salvage typically involves a multifaceted approach, including the following:

Revascularization procedures: Limb salvage often starts with revascularisation procedures, such as angioplasty, stenting, or bypass surgery. These interventions restore blood flow to the affected limb, addressing the underlying vascular problem and promoting tissue viability [54].

Wound care: Comprehensive wound care is crucial in limb salvage. This includes meticulous cleaning, necrotic tissue debridement, and specialised dressings to create an optimal environment for healing [55].

Surgical interventions: Surgical procedures may be necessary to remove necrotic tissue, reshape the limb, or repair damaged structures. These interventions aim to optimise functional recovery chances [56].

Limb salvage offers numerous advantages to patients. It enables them to maintain their independence, perform daily activities, and avoid the physical and psychological challenges associated with limb loss. Patients who undergo successful limb salvage often experience improved overall well-being and a higher quality of life.

Amputation

In cases where limb salvage is not medically viable or where the risks associated with continuing to preserve the limb outweigh the benefits, amputation may become necessary. Amputation is a carefully considered decision to prevent further complications, such as sepsis, life-threatening infections, or unmanageable pain. The choice of the level of amputation is critical and is determined by balancing the removal of necrotic tissue with the preservation of functional limb length [57].

Modern prosthetic technology has significantly improved the quality of life for individuals who undergo amputation. Prosthetic limbs are designed to replicate natural movement and functionality, allowing amputees to regain mobility and independence. The rehabilitation process post-amputation, which includes physical therapy and prosthetic fitting, is crucial in helping individuals adapt to their new circumstances and achieve the highest level of functionality possible [58].

Quality of Life Considerations

Quality of life is a pivotal consideration in managing gangrene in individuals with PVD, regardless of whether the approach involves limb salvage or amputation. Recognising and addressing the multifaceted aspects of quality of life is integral to the comprehensive care of these patients:

Limb salvage: Successful limb salvage not only preserves the physical function of the affected limb but also exerts a profound positive influence on the psychological well-being of patients. Maintaining independence, self-esteem, and positive body image is crucial for overall quality of life. When limb salvage is achieved, individuals can continue to engage in daily activities, pursue their hobbies and interests, and maintain their roles within their families and communities. This preservation of physical function and autonomy significantly contributes to an improved quality of life [59].

Amputation: While amputation involves the loss of a limb, advancements in prosthetic technology have substantially improved the functional outcomes and quality of life for individuals who undergo this procedure. Modern prosthetic limbs are designed to provide remarkable mobility and functionality, enabling amputees to lead active lives. Rehabilitation and support services are integral components of post-amputation care, facilitating the adaptation to new circumstances. Physical therapy and prosthetic training are essential to help individuals regain mobility and independence, enhancing their quality of life [60].

Psychosocial support: The emotional and psychological aspects of living with gangrene or limb loss cannot be underestimated. These challenges can significantly impact a patient's quality of life. A multidisciplinary approach to care, including counselling and emotional support, is vital in addressing these psychosocial aspects. Patients benefit from guidance and counselling to cope with the emotional toll of their condition, adapt to changes in body image, and develop strategies for managing stress and anxiety [61].

Vocational rehabilitation: Maintaining gainful employment and vocational satisfaction is integral to the quality of life. Vocational rehabilitation programs assist individuals in re-entering the workforce or adapting to new occupational roles post-amputation. These programs offer valuable support in career counselling, skill development, and workplace accommodations, ensuring that individuals can continue to pursue fulfilling and meaningful careers [62].

Future directions and research

Advances in PVD Treatment

Ongoing research is driving the exploration of innovative and promising avenues for treating PVD, which extends to managing complications such as gangrene. These potential future developments hold the potential to revolutionise PVD treatment by offering new modalities that enhance patient outcomes and quality of life:

Drug therapies: Research efforts are increasingly focused on developing novel medications tailored to address specific aspects of PVD. These medications aim to target atherosclerosis, inflammation, and vascular function with greater precision. The emergence of more effective drug therapies could provide non-surgical management options for PVD patients, particularly those in the early stages of the disease. These pharmaceutical interventions may better control underlying factors contributing to PVD progression and complications such as gangrene [63].

Gene therapies: A burgeoning field in medical research, gene-based therapies show immense promise in promoting vascular repair and regeneration. In the context of advanced PVD, gene therapies may be vital in stimulating the growth of new blood vessels, a process known as angiogenesis. Gene-based therapies could offer innovative solutions for patients with severe ischemia by harnessing the body's regenerative capabilities, ultimately reducing the risk of gangrene development and limb loss [64].

Minimally invasive interventions: Advances in endovascular techniques continue to shape the landscape of PVD treatment. Innovations such as drug-eluting devices and bioresorbable stents are on the horizon, offering the potential for less invasive and more targeted interventions. These technologies may expand the scope of minimally invasive treatments for PVD, allowing for improved blood flow restoration and reduced procedural risks. Minimally invasive approaches have the potential to play a crucial role in preventing gangrene by addressing vascular blockages earlier and more effectively [65].

Biologics and stem cell therapies: Research into biologics and stem cell therapies for promoting angiogenesis and tissue regeneration in ischemic limbs is an exciting frontier in PVD treatment. These therapies aim to harness the body's healing mechanisms by introducing growth factors, stem cells, or other biologically active substances. By enhancing blood vessel growth and tissue repair, biologics and stem cell therapies can mitigate the effects of PVD, reduce the risk of gangrene, and improve overall limb function and quality of life for affected individuals [66].

Biomarkers for Early Detection

Early detection of PVD and its complications, including gangrene, is paramount for timely and effective intervention. Emerging research endeavours are increasingly focused on identifying specific biomarkers to aid in the early diagnosis and risk assessment of PVD-related gangrene. This proactive approach holds promise for improving patient outcomes and reducing the severity of the disease. Here, we delve into the potential of biomarkers and advanced imaging techniques in the early detection of gangrene within the context of PVD [67].

Biomarkers for risk assessment: Biomarkers are measurable biological indicators that can provide valuable insights into certain diseases' presence, progression, or risk. In the context of PVD and gangrene, researchers are exploring various biomarkers that may offer early detection and risk assessment capabilities. Among the potential biomarkers of interest are markers of inflammation, which play a pivotal role in the pathogenesis of PVD. Elevated levels of inflammatory markers, such as CRP and pro-inflammatory cytokines, may be early warning signs of vascular dysfunction. Additionally, markers of endothelial dysfunction, such as vascular cell adhesion molecules (VCAM-1) and endothelial nitric oxide synthase (eNOS), could offer valuable insights into early vascular changes that precede gangrene development. Genetic factors are also scrutinised, as specific genetic variations may increase an individual's susceptibility to PVD-related complications. Identifying these biomarkers holds promise for risk stratification, allowing healthcare providers to intervene earlier in high-risk individuals [68].

Advanced imaging techniques: Besides biomarkers, advanced imaging techniques are emerging as non-invasive tools for the early detection of vascular changes that may lead to gangrene. For instance, molecular imaging allows visualising specific molecular processes within blood vessels. This innovative approach can detect early signs of vascular inflammation, plaque formation, and blood flow abnormalities. Molecular imaging techniques, such as positron emission tomography (PET) and magnetic resonance imaging (MRI) with contrast agents targeted to specific molecules, enable healthcare providers to assess vascular health and identify potential risk factors for gangrene at an earlier stage. These non-invasive imaging modalities have the potential to enhance diagnostic accuracy and guide personalised treatment strategies [69].

Rehabilitation and Prosthetic Innovations

Innovations in rehabilitation and prosthetic technology are poised to significantly enhance the quality of life for individuals who have undergone amputation due to gangrene. These advancements not only hold promise for improving mobility and functionality but also offer a more seamless integration of prosthetic limbs with the user's body and provide crucial psychosocial support:

Prosthetic advancements: Ongoing research and development in prosthetic design and materials are expected to yield more advanced and comfortable prosthetic limbs. These innovations aim to enhance both the functional capabilities and user experience. Prosthetic limbs are becoming lighter, more durable, and better adapted to various activities, allowing individuals to lead more active and fulfilling lives. Furthermore, advancements in socket design, which is the interface between the residual limb and the prosthesis, are improving comfort and stability, reducing issues such as skin irritation and discomfort [70].

Sensory feedback: An exciting frontier in prosthetic technology is the development of prosthetic devices with sensory feedback capabilities. These innovations aim to bridge the gap between the user and the prosthetic limb, enabling more natural control and sensory perception. Research in this area explores technologies that can provide users with tactile sensations and proprioception, allowing them to feel and interact with their prosthetic limbs more intuitively. This sensory feedback can be a game-changer in restoring a sense of wholeness and improving functionality [71].

Rehabilitation protocols: Rehabilitation protocols for individuals with gangrene or limb loss continually evolve to address their unique needs. Advances in virtual reality (VR) and robotics are being integrated into rehabilitation programs to enhance engagement and outcomes. VR-based rehabilitation can simulate real-world scenarios, allowing individuals to practice mobility and adapt to their prosthetic limbs in a safe and controlled environment. Robotics, such as exoskeletons, can assist in gait training and help individuals regain strength and mobility more rapidly. These technologies can potentially expedite the rehabilitation process and improve the overall functional outcomes for amputees [72].

Psychosocial support: Coping with limb loss due to gangrene can present significant psychological and emotional challenges. Research in psychosocial support strategies is a continuing priority. Psychologists and counsellors are developing interventions addressing the emotional well-being of individuals with limb loss. Support groups and peer mentoring programs provide opportunities for individuals to share experiences and receive guidance from others who have gone through similar challenges. Furthermore, telehealth and digital platforms are expanding access to mental health support, making it more accessible to those in need [73].

## Conclusions

In conclusion, gangrene within the context of peripheral vascular disease represents a complex and severe medical condition that demands our attention and understanding. This review has underscored the importance of timely intervention in diagnosing and managing gangrene, emphasising the need for swift and appropriate treatments to preserve limbs and prevent life-threatening complications. It has also highlighted the significance of multidisciplinary care, recognising the pivotal role of healthcare professionals from diverse fields in delivering comprehensive patient-centred care. Looking forward, we call for heightened awareness among healthcare providers and the general public, further research to advance our knowledge and treatment options, and a continued commitment to improving the quality of life for those affected by gangrene in peripheral vascular disease. By fostering collaboration, raising awareness, and advancing research, we can aspire to better outcomes and brighter prospects for individuals confronting this challenging medical condition.
